# Krüppel-like Factor 2 (KLF2) in Immune Cell Migration

**DOI:** 10.3390/vaccines9101171

**Published:** 2021-10-13

**Authors:** Jens Wittner, Wolfgang Schuh

**Affiliations:** Division of Molecular Immunology, Department of Internal Medicine III, Nikolaus-Fiebiger-Center, University Hospital Erlangen, Friedrich-Alexander University Erlangen-Nürnberg, 91054 Erlangen, Germany; jens.wittner@uk-erlangen.de

**Keywords:** Krüppel-like factor 2, KLF2, migration, S1PR1, B cells, T cells, plasma cells, monocytes, neutrophils, NK cells

## Abstract

Krüppel-like factor 2 (KLF2), a transcription factor of the krüppel-like family, is a key regulator of activation, differentiation, and migration processes in various cell types. In this review, we focus on the functional relevance of KLF2 in immune cell migration and homing. We summarize the key functions of KLF2 in the regulation of chemokine receptors and adhesion molecules and discuss the relevance of the KLF2-mediated control of immune cell migration in the context of immune responses, infections, and diseases.

## 1. Introduction

Krüppel-like factor 2 (KLF2), a member of the Krüppel-like factor family of transcription factors, is characterized by a zinc-finger-containing DNA binding domain. The name of the family is based on the German word “Krüppel” for “cripple” and describes the phenotype of a Drosophila loss of function mutant with an abnormal segmentation in the abdominal region of the larvae [1]. The Drosophila Krüppel transcription factor belongs to the so-called gap genes, a group of genes crucial for the development of the Drosophila larvae [1]. The family of Krüppel-like factors in vertebrates consists of 17 members which all play important roles in the differentiation processes and proliferation control, as well as cell adhesion and migration in various cell lineages in vertebrates [2]. In this article, we focus on the functional implications of KLF2 in immune cell migration and adhesion. KLF2 was discovered by Anderson et al. (1995) and named lung KLF (LKLF) because of its high expression in lungs [3]. Anderson et al. already reported a high KLF2 abundance in the spleen. KLF2 is expressed in the endothelial cells, subsets of the B cell and the T cell lineage, NK cells, monocytes, and macrophages, as well as in neutrophils. The expression of KLF2 within a certain cell lineage, such as the B and T cell lineage, varies and correlates with the differentiation and activation status [4]. The importance of KLF2 was demonstrated in 1997, when Kuo et al. generated a genomic knockout mouse model for KLF2 and reported that KLF2-deficient embryos died between day E12.5 and E14.5 due to hemorrhaging, with defective blood vessels and an abnormal tunica media [5]. These findings revealed the crucial role of KLF2 in embryonic development and endothelial cell biology. Moreover, KLF2 was implicated in mast cell physiology and adipogenesis, as well as diseases such as atherosclerosis, thrombosis and lymphomas (e.g., Marginal zone B cell lymphoma) [2,6,7,8,9,10].

## 2. KLF2—A Central Regulator of Quiescence and Activation

KLF2 is involved in a variety of regulatory pathways including immune cell quiescence, proliferation, and activation, as well as adhesion and migration [4]. During T cell development in the thymus, KLF2 is upregulated in single-positive CD4^+^ and CD8^+^ T cells. Upon T cell activation, KLF2 is rapidly downregulated [11]. The investigation of peripheral T cell populations in KLF2^-/-^/Rag2^-/-^ chimeric mice revealed spontaneous T cell activation and increased cell death by FAS-mediated apoptosis, suggesting that, on the one hand, KLF2 regulated T cell quiescence and, on the other hand, served as a survival factor. Moreover, KLF2-deficient peripheral T cells displayed an enhanced expression of surface activation markers [11]. The ectopic expression of KLF2 in Jurkat T cells resulted in a quiescent phenotype with a decreased proliferation, reduced cell size and protein synthesis, as well as a decreased expression of activation markers such as CD71 (transferrin receptor). Ectopic KLF2 expression correlated with a reduction in c-myc expression [12]. Furthermore, doxycycline-induced expression of KLF2 in Jurkat T cells resulted in the inhibition of proliferation and DNA synthesis. Mechanistically, KLF2 bound to the p21^WAF1/CIP1^ promoter, and thus directly regulated p21 expression [13]. Additionally, in the IL-2-dependent human T cell lymphoma cell line Kit225, the demethylation of the promoter for human telomerase reverse transcriptase (hTERT) resulted in the direct binding of KLF2 to the CpG sequences in the hTERT promotor, inhibiting the hTERT gene expression, and thus keeping the cells in the resting phase [14]. In summary, KLF2 interferes with T cell proliferation by suppressing c-myc and activating the cell cycle inhibitor p21, as well as the direct repression of the cell cycle regulatory gene, hTERT. 

In developing B cells, KLF2 was identified as a pre-B cell receptor (pre-BCR)-controlled target gene by our group using the tetracycline-controlled induction of the pre-BCR [15]. The upregulation of KLF2 as a consequence of pre-BCR signaling involves Erk5 activation and the subsequent phosphorylation of the transcription factors, mef2c/2d. Phosphorylated mef2c/2d activates KLF2 expression and, in parallel, activates the expression of immediate early genes such as Egr1/2, Ier2, Jun/Fos. KLF2 abundance increased over time and resulted in the suppression of the immediate early genes. Through this negative feed-forward loop, pre-B cell expansion is terminated. In parallel, mef2c activates Egr and IRF-4 to foster pre-B cell differentiation to immature B cells [16]. In 2000, Glynne et al. attempted to identify factors involved in the regulation of quiescence versus proliferation in B cells, as well as in tolerance, by comparative gene expression profiling. Among the genes that were highly expressed in naive B cells and reduced upon stimulation, they found members of the Krüppel-like family, including KLF2 [17]. The high expression of KLF2 in naive B cells was confirmed in various studies using either qPCR or flow cytometric analyses [18,19,20,21,22]. The activation of the B cell receptor (BCR), as well as LPS and anti-CD40/IL-4 activation of naïve B cells led to the downregulation of KLF2 [19,20,21]. In proliferating pro-B and activated splenic B cells, the ectopic expression of KLF2 using retroviral constructs resulted in impaired proliferation of both pro-B cells and LPS-activated blasts. The underlying mechanisms included the downregulation of c-myc and the upregulation of p21. KLF2 overexpression also impaired LPS-mediated B cell activation [20]. Therefore, the KLF2-mediated control of proliferation versus quiescence employs the same regulatory pathways in T and B cells. Moreover, experimental data by Bhattacharya et al. suggested that KLF2 might also function as a pro-survival factor in in vitro activated B cells [23].

Furthermore, KLF2 is involved in the regulation of the activation in myeloid cells. KLF2 is highly abundant in resting monocytes and is rapidly downregulated upon stimulation with LPS [24]. These findings were in line with the observed mechanisms in B and in T cells. In summary, KLF2 is crucial for the resting/quiescent state in T, B and myeloid cells and inhibits the proliferation of T and B cells in a c-myc-dependent manner. The downregulation of KLF2 is a prerequisite for myeloid cell as well as T and B cell activation. Furthermore, KLF2 also exerts pro-survival functions in T and B cells.

## 3. Requirements for Immune Cell Migration and Homing

In this review, we focus on the role of KLF2 in immune cell migration and adhesion in immune cell homeostasis, as well as in immune cell migration during infections and under disease conditions. The migration behavior of immune cells depends on several factors such as the activation and maturation status of the cell and the environmental context (e.g., tissue-specific chemokines, inflammation, infection). For example, T cells activated by Peyer’s Patch (PP)-derived, CCR6-expressing dendritic cells (DC) [25,26], are imprinted for intestinal homing by expressing integrin (Itg) α4β7, as well as CCR9 [27]. Furthermore, the imprinting of immune cells is also driven by the stromal cell environment. Mesenteric lymph node (mLN) stromal cells, but not peripheral lymph node (pLN) stromal cells, express retinoic acid-producing enzyme, RALDH2, and thus the primed T cells express CCR9 for gut mucosal migration [26,28].

As immune cells migrate through the vascular system, the process of tissue entry to the lymph nodes (LN) is regulated by three steps of extravasation. High endothelial venules (HEVs) are the entry site to lymph nodes for immune cells from the blood. HEVs are present in all lymphoid organs except the spleen [29,30]. The first step of extravasation, the tethering and rolling process along the HEVs, is induced by selectin/addressin interaction of immune cells with endothelial cells [29,30]. The second step of extravasation is the chemokine-mediated recognition of the appropriate extravasation site. Chemokines, such as CCL21, CXCL12 or CXCL13, are presented on the vascular side of the HEVs by glycosaminoglycans (GAGs). The recognition of these cytokines requires the expression of CCR7 (receptor for CCL21), CXCR4 (receptor for CXCL12) or CXCR5 (receptor for CXCL13) on the immune cell surface. Next, chemokine-mediated signals activate integrins on the immune cell surface leading to a conformational change of the integrin structure from an inactive to an active conformation [31]. In the third and final step, lymphocytes stop at the correct site for extravasation, mediated by the attachment of specific integrins to intercellular adhesion molecules (ICAM) expressed on the HEV surface. This three-step process is followed by the transcellular or paracellular transmigration of the immune cell through the endothelium into the tissue of destination [30]. Tissue-specific homing of immune cells is guided by the expression of specific combinations of chemokine receptors and integrins on the surface of immune cells which allow the sensing of chemokine gradients in tissues.

Specifically, lymphocyte homing to LN requires L-selectin (CD62L), LFA-1 (αLβ2 integrin) and the chemokine receptor, CCR7 (receptor for CCL19 and CCL21). Inside the LN, T cells migrate to the T cell zone following the gradients of CCL19 and CCL21, whereas B cells are directed to the B cell follicles following a CXCL13 gradient [32]. For homing to PP, the expression of Itgα4β7, LFA1, and L-selectin on the immune cell surface is required [33]. Homing to the gut lamina propria (LP) involves Integrin α4β7 combined with CCR9 expression on the cell surface on B and T lymphocytes. In addition, P-selectin was described to be important for Th1 cell migration to the gut LP [34]. Furthermore, the CCL28 chemokine receptor CCR10 was described to be indispensable for IgA-secreting plasmablasts (PB) migration to the mucosal sides in a genomic CCR10 knock-out mouse strain [35]. In their study, Morteau et al. demonstrated a striking reduction in IgA-secreting plasma cells (PC) in the mammary gland but, interestingly, minor effects on IgA^+^ PC abundance in the LP of the gut, including the small and large intestine [35]. Homing to non-intestinal mucosal tissues of the cutaneous lymphocyte antigen (CLA)-expressing IgA B mem is driven by E-selectin binding such as in chronically inflamed bronchial or oral mucosal tissue [36].

The entry of immune cells to the bone marrow (BM) requires Itgα4β1 and CXCR4 expression. BM entry is facilitated by the bone marrow microvasculature consisting of special sinusoids with a large diameter and a decreased blood flow, as well as a fenestrated endothelium [37,38,39,40,41]. Homing to sites of inflammation is mediated by the chemokine receptor CXCR3 and cell activation through its ligands: CXCL9, CXCL10 and CXCL11. In T cells, the cell fate decision of cytotoxic CD8^+^ T cells, as well as CD4^+^ Th1 cell fate is highly linked to CXCR3 expression [42]. In this context, classical Th2 cells are capable of chemokine receptor-independent tissue scanning by the expression of αVβ3 integrin [43]. In B cells, CXCR3 expression is linked to the infiltration of B cells into inflamed tissues of rheumatoid arthritis. Furthermore, innate lymphocytes such as NK and NKT cells express CXCR3 to migrate to inflammatory sites as a first-line defense [44,45]. In CD8^+^ T cells, KLF2 is shown to negatively regulate CXCR3 expression and pro-inflammatory responses to CXCL10. Here, KLF2 abundance differentially regulates T cell responses by the strength of its expression [46]. Furthermore, pro-inflammatory migration to the gut in ulcerative colitis is mediated by integrin α4β7 for regulatory and effector T cells, while Treg uniquely require additional GPR15 expression to enter the inflamed tissue [47]. During inflammation, T effector cells enter the skin, gut, lung or salivary glands. To ensure a better protection against reinfection, not all lymphocytes leave the infected tissue after pathogen clearance. The tissue-resident memory CD8^+^ T cells express CD103 and downregulate S1PR1 and CCR7 expression to prevent their egress and ensure long-term residency [48]. B cell migration to the inflamed sides of patients suffering from Lyme neuroborreliosis and into the B cell-rich cerebrospinal fluid is mainly controlled by CXCR5-mediated migration of B cells to CXCL13 [49]. In skin inflammation, IL10^+^ regulatory B cells (Breg) need α4β1 integrin to enter the inflamed skin [50].

The migration of immune cells from tissues (e.g., T cell exit from the thymus, plasmablast (PB) exit from spleen) to the blood is controlled by sphingosine-1-phosphate (S1P) and its receptors (S1PR) [51,52]. In the next chapter, we discuss the impact of KLF2 on migration and homing of T cell subsets, B cells and plasma cells, myeloid cells, and NK cells.

## 4. KLF2 and T Cell Migration 

**Figure 1:**
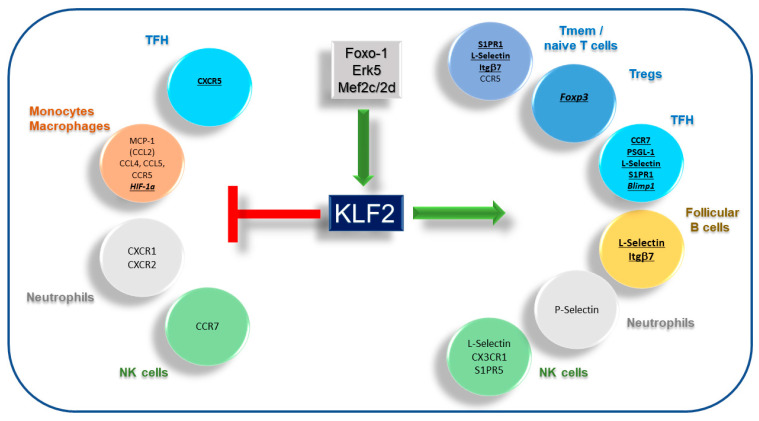
Regulation of chemokine receptors, adhesion molecules and selected transcription factors in immune cells by KLF2. KLF2 expression is activated by signaling cascades involving Foxo-1, Erk5 and mef2c/2d in various immune cells. Genes negatively regulated by KLF2 (left side of the scheme): CXCR5 in TFH cells, MCP-1 (CCL2), CCL4, CCL5, CCR5 and HIF-1α in monocytes and macrophages. CXCR1 and CXCR2 in neutrophils and CCR7 in NK cells. Genes positively regulated by KLF2 (right side of the scheme): S1PR1, L-Selectin, Itgβ7, CCR5, as well as CCR7 and PGSL-1 and Foxp3 in different T cell subsets. L-Selectin and Itgβ7 in follicular (FO) B cells. P-Selectin in neutrophils. L-Selectin, CX3CR1 and S1PR5 in NK cells. Genes directly regulated by KLF2 are written in bold letters and are underlined. Transcription factors in italics. NK cells, natural killer cells; TFH, T follicular helper cells; Treg, regulatory T cells; NK, Natural killer cells; Itg, integrin.

**Figure 2:**
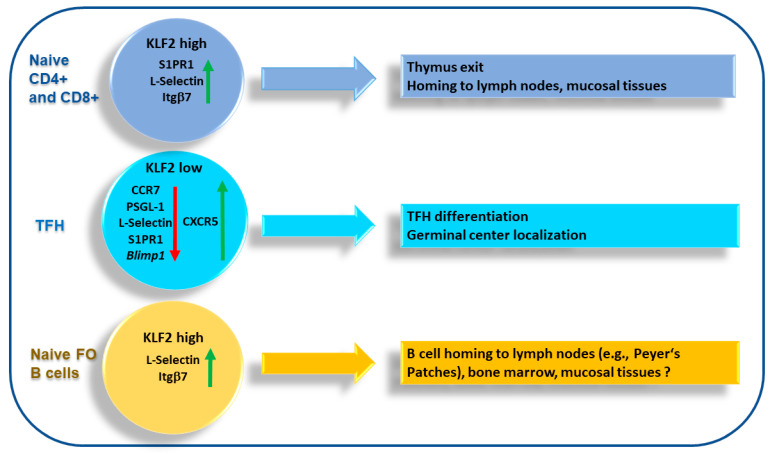
KLF2-mediated regulation of the differentiation and migration of naïve T cells, TFH cells and naïve B cells. In naïve T cells, KLF2 induces the upregulation of S1PR1, L-Selectin and Itgβ7. In TFH cells, KLF2 is downregulated to low levels (by ICOS/ICOSL signals). Downregulation of KLF2 in TFH cells results in upregulation of CXCR5 and downregulation of Blimp1, CCR7, PSGL-1, S1PR1 and L-Selectin. In naïve FO B cells, KLF2 regulates L-Selectin and Itgβ7 expression which might affect B cell migration to lymph nodes, bone marrow and mucosal tissues. Downregulation is indicated by red arrows; upregulation is indicated by green arrows.

Upon T cell development in the thymus, naïve CD4^+^ and CD8^+^ T cells leave the thymus and migrate to secondary lymphoid organs via the blood. The thymus exit is controlled by Sphingosine-1-phosphate (S1P), a bioactive sphingolipid metabolite which is highly abundant in the blood. S1P is sensed by S1P receptors (S1PR) 1-5, resulting in the exit or entry of migrating cells depending on the expressed S1PR [53]. As described above, S1PR1 is essential for thymus exit of mature naïve T cells [51,54]. The first relation between cell migration via S1PR and KLF2 was demonstrated by showing that KLF2-deficient T cells fail to leave the thymus due to the lack of S1PR1 expression [51]. Developing T cells upregulate the S1P1R which renders them susceptible to S1P gradients and attracts them to the blood. Carlson et al. used ChIP analysis to demonstrate that S1PR1 is controlled by direct binding of KLF2 to the S1PR1-promoter [55]. Using KLF2-deficient mice, Carlson et al. revealed that thymocytes accumulated inside the thymus due to the impaired upregulation of S1PR1 and, as a consequence of a defective exit, peripheral CD4^+^ and CD8^+^ T cell subsets were virtually absent (Figure 1 and Figure 2) [55]. On the other hand, S1PR1 is necessary and sufficient for the persistence of activated CD4^+^ T cells in inflamed tissues. In inflammatory models, activated CD4^+^ T cells with enhanced S1PR1 showed a higher motility inside the inflamed tissue but were not impaired in their migration to the draining LN [56]. Additionally, T effector cells downregulated S1PR1 as well as CCR7 in skin inflammation, to ensure a persistence inside the inflamed tissue [57]. These regulations make S1PR1 and its regulators (such as KLF2 for S1PR1) interesting therapeutic targets for the treatment of chronic inflammations. The migration of mature, naïve T cells from the thymus via the bloodstream to peripheral LN is driven by S1PR1 and L-Selectin, which are both direct target genes of KLF2 [55,58]. In addition to its crucial role for thymus exit of T cells, KLF2 also controls differentiation processes and cell fate decisions. T cells which express low levels of KLF2 differentiate into regulatory T cells or CD8^+^ T cells, whereas classical CD4^+^ T helper cells express higher levels of KLF2 [59].

### 4.1. T Cell Activation

While KLF2 is highly expressed on naïve mature CD4^+^ and CD8^+^ T cells, KLF2 is repressed upon T cell activation [11,60]. Takada et al. analyzed KLF2 function in CD8^+^ T cells using a conditional CD4-Cre KLF2fl/fl mouse model, inducible KLF2 deletion upon T cell receptor (TCR) activation, and KLF2 re-expression in post-activated CD8^+^ T cells. They confirmed S1PR1 and L-selectin regulation by KLF2 and the deregulated migration of KLF2-deficient T cells. The proliferation, however, was not impaired [60]. In a study by Preston et al., the activation of the TCR on CD8^+^ T cells led to the downregulation of KLF2, L-Selectin, S1PR1, CCR2, Itgα4, F2rt1, Itgβ2, CD44, Itgβ7, and Itgα1, but the upregulation of CXCR3, Fut4 and CCR4, which enabled CD8^+^ T cells to enter inflamed tissues [46]. Downregulation of KLF2 was required for the expansion and tissue positioning of effector T cells. Post-activated CD8^+^ T cells re-expressed KLF2 to enable their migration via S1PR1 and L-Selectin, allowing memory T cells to travel through lymphoid tissues. [46,60]. In contrast to quiescent T cells, in which KLF2 is highly expressed, CD8^+^ T cells that are activated through their TCR need to proliferate and express the inflammatory chemokine receptor CXCR3 to respond to CXCL10. Both, proliferation and CXCR3 expression are induced by KLF2 downregulation in a gradual response, determined by the affinity of the TCR ligand (i.e., TCR receptor signaling strength) followed by an integrated activation of PI3K/protein kinase B (PKB), as well as MAP kinases ERK1/2 [46]. KLF2 downregulation is regulated by the strength and duration of TCR and cytokine stimulation. It is important to mention that, in addition to the TCR signal strength, the source of T cell activation determines the subsequent migration and homing of T cells. For example, CD8^+^ T cell stimulation by CD103^+^ mucosal dendritic cells, causes T cells to migrate to the mucosal inductive (i.e., Peyer’s Patches) or effector (i.e., Lamina Propria) sites by Itgα4β7 and CCR9 [27]. In this context, KLF2 plays a major role, as it is capable to directly target the Itgβ7 promotor [61].

The role of KLF2 in CD4^+^ T cell subsets was extensively studied by numerous groups. Sebzda et al. reported that KLF2-deficient CD4^+^ T cells showed an abnormal migration with homing to non-lymphoid tissues (such as liver and muscle), because the deletion of KLF2 caused the dysregulation of various chemokine receptors (e.g., the upregulation of CCR5, CXCR3, CXCR5) in KLF2-deficient CD4^+^ T cells [62]. However, these findings remain controversial as, for example, Weinreich et al. claimed a non-autonomous cell effect of CXCR3 upregulation in KLF2-deficient T cells. In mixed bone marrow chimeras with KLF2 deficient cells, the upregulation of CXCR3 was also found on wildtype bystander cells. The reason for this finding was that KLF2-deficient T cells secreted more IL-4 which in turn led to the upregulation of CXCR3 [63].

### 4.2. T Follicular Helper Cells

T follicular helper (TFH) cells are a specialized subset of CD4^+^ T cells that are critically important for the formation of germinal centers (GC) and for promoting B cell activation by the direct interaction with B cells and secretion of IL-21 to foster B cell proliferation. TFH cells are characterized by the expression of the transcription factor, B cell lymphoma 6 (Bcl6) and by the cell surface markers CXCR5, PD1 and inducible T-cell costimulator (ICOS). The proper positioning of TFH cells in the B cell follicle is required for the generation of a functional GC and is driven by ICOS signals resulting in Foxo1-mediated downregulation of KLF2 [64].

During TFH cell differentiation, the transcription factor Blimp1 (encoded by the prdm1 gene) needs to be downregulated to allow the upregulation of Bcl6. Besides Blimp1 downregulation, S1PR1 and KLF2 are also repressed in differentiating TFH cells. The prdm1 gene, but not the Bcl6 gene is shown to be a direct target gene of KLF2 in CD4^+^ TFH cells by ChIP analysis [65]. Low abundances of KLF2 during differentiation might be responsible for low Blimp1 levels, but do not affect Bcl6 expression directly. Low Blimp1 levels, in turn, lead to the upregulation of Bcl6 [64]. ICOS/ICOSL signals were shown to downregulate Blimp1, T-bet, CCR7 and KLF2, while the downregulation of KLF2 directly resulted in the upregulation of CXCR5 and downregulation of the positioning factor CCR7, which regulates lymph node entry of both B and T cells from the blood [66]. The downregulation of KLF2 expression in TFH cells is a key feature of early TFH cells leaving the B/T cells border and migrating inside the B cell follicle where CXCL13, the ligand of CXCR5, is produced. Therefore, low KLF2 expression enables the expression of CXCR5 and reduces the expression of CCR7 and PSGL-1, a ligand for P-Selectin (Figure 1 and Figure 2). Of note, the direct binding of KLF2 to the CXCR5, and to the CCR7 and PSGL-1 genes/promoters was observed [66]. These findings demonstrate that KLF2 is not a simple “on/off” switch but rather modulates the expression of its target genes in a dose-dependent manner and can activate or repress the transcription of its target genes. Activatory or inhibitory functions of KLF2might depend on the cofactors and interaction partners of KLF2.

### 4.3. Regulatory T Cells

Regulatory T cells (Tregs) are a specialized CD4^+^ T cell subset involved in the maintenance of tolerance, the limitation of inflammatory processes and the prevention of autoimmunity. Tregs exert their regulatory effects by secreting suppressive cytokines, cytolysis, metabolic deprivation, and by targeting dendritic cell differentiation and activation. Tregs are characterized by the expression of the key transcription factor Foxp3 [67] and can be generated either in the thymus (tTreg) or induced in the peripheral tissues (iTregs) [68]. The stabilization of KLF2, i.e., the prevention of KLF2 protein degradation, resulted in an increased generation of iTregs but not tTregs. Furthermore, the direct binding of KLF2 to the Foxp3 promoter was observed and Treg-specific deletion of KLF2 using a Foxp3-cre deleter resulted in the onset of autoimmune diseases (Figure 1, Table A1 in Appendix A) [59,69]. In human tonsils, CD69^−^ and CD69^+^ Tregs were described. Compared to the spleen, CD69^+^ Tregs are more frequent in tonsils. These CD69^+^ Tregs displayed a tissue-resident phenotype with a low expression of KLF2 and S1PR1, but enhanced CTLA-4 expression and enhanced suppressive regulatory functions mediated by CD39 expression and ICOS induction, resulting in IL10 production [70]. Thus, Treg tissue retention is controlled by KLF2 and S1PR1 downregulation, a mechanism also utilized by memory T cells. (as described in the following paragraph).

While KLF2 is downregulated in activated T cells, post-activated T cells re-express KLF2 to enable trafficking via S1PR1 and L-Selectin and the establishment of a CD8^+^ T cell memory compartment in lymphatic tissues [60,71]. It is known that especially CD8^+^ T memory cells circulate through non-lymphatic tissues such as the skin, to screen the tissue for pathogens and ensure their fast detection and immune response [72]. Surprisingly, T memory cells in non-lymphatic tissues express low levels of KLF2. It is suggested that local cues from the non-lymphatic environment (including TGF-β, IL-33, TNF) cause a decrease in KLF2 and a subsequent decrease in S1PR1 to support the tissue retention of Tmem [71]. Furthermore, memory CD4^+^ T cells are described to be most abundant in many non-lymphoid tissues. They are the predominant cell type observed exiting the skin via the lymphatics which indicates that many memory CD4^+^ T cells recirculate [73]. This observation aligns with the finding that KLF2 expression is higher in CD4^+^ compared to CD8^+^ T cells, and thus S1PR1 is maintained on the surface of CD4^+^ Tmem cells. Stable populations of Tmem cells remain present in the peripheral organs after the clearance of an infection due to KLF2 downregulation, leading to an active form of CD69 which blocks S1PR1 function by conformational changes due to CD69/S1PR1 binding and an inhibition of S1PR1 expression [72]. Furthermore, the decision of an effector T cell to differentiate into an epithelium-resident memory T cell is based on the downregulation of KLF2, S1PR1, L-Selectin, CCR7, eomesodermin (EOMES), killer cell lectin like receptor G1 (KLRG1) and T-bet together with an upregulation of CD69 and CD103. The subset of T effector memory cells that recirculate and exit the tissue via the lymphatic vessels are driven by KLF2 expression together with S1PR1 and CCR7, in combination with CD69 and CD103 downregulation [73].

Of note, γδ T cells, a subset of T cells required, e.g., in the gut and other mucosal tissues, develop in the thymus [74] and upregulate S1PR1 expression, similar to αβ T cells. KLF2-deficiency results in their accumulation in the thymus due to their defective exit [75]. Hence, KLF2 plays a functional role in all major T cell subsets by controlling thymic exit, peripheral migration and homeostasis.

### 4.4. KLF2 and T Cells in Diseases 

Infection of lymphocytes with the HI virus-1 (HIV-1) results in the deregulation of many intrinsic cellular programs in order to prevent the cells’ antiviral immune responses. For entry into human CD4 T cells, the HIV-1 R5 strain utilizes CCR5 as a co-receptor to target the host cell. In resting, but not CD3/CD28-activated primary human CD4^+^ T cells, KLF2 directly binds to the CCR5 promoter, resulting in CCR5 expression (Figure 1, Table A1) [76]. In addition, a strong correlation between the activation status of CD4^+^ T cells, in which a direct regulation of CCR5 in resting cells by KLF2 was demonstrated, and the susceptibility of an HIV-1 (R5) infection was observed. Aside from virus entry via KLF2-regulated CCR5 into naïve CD4^+^ T cells, HIV-1 infection itself represses Foxo-1 activity and KLF2 expression, resulting in the downregulation of L-Selectin (CD62L) [77]. In addition, S1PR1 mRNA expression decreased as a result of KLF2 downregulation, which together with an enhanced expression of CD69 (a S1PR1 suppressor) resulted in the defective migration of CD4^+^ T cells [77]. These findings propose a scenario in which HIV-1-infected, naïve CD4^+^ T cells proliferate due to the loss of KLF2-mediated cell cycle repression. This might help the virus to reproduce more efficiently, and, in addition, infected T cells might lose their ability to migrate properly (Table A1).

As mentioned earlier, KLF2 is important for the generation of iTRegs. Along this line, KLF2 binding to the Foxp3 promoter was demonstrated by ChIP analyses [59]. As KLF2 is involved in the homeostasis of Tregs, the dysfunction of KLF2 could result in autoimmune diseases. Indeed, Pabbisetty et al., by deleting KLF2 in Tregs using a Foxp3-Cre mouse model, found that the deletion of KLF2 in Tregs resulted in signs of psoriasis and signs of inflammatory bowel disease [69].

Furthermore, KLF2 is implicated in the regulation of TFH-mediated Type1 Diabetes (Table A1). In this context, KLF2 was identified as a target of microRNA-92a, a microRNA regulating the sizes of the TFH precursor pool [78]. In allergic asthma, Th2 cells play a major role by infiltrating the lung and by reacting to inhaled allergens. Tindemans et al. revealed a Notch-dependent effect on lung trafficking of Th2 cells. While Notch-deficient Th2 cells were prevented from invading the lung in a house dust mite driven, eosinophilic airway inflammation model, these Th2 cells could not leave the lymph node due to a lack of KLF2 and its downstream target S1PR1. They concluded, that in allergic airway inflammation, the Notch signaling pathway led to a KLF2-regulated, S1PR1-mediated egress of Th2 cells from the LN (Table A1) [79].

## 5. KLF2 and B Cell Migration

KLF2 is differentially expressed during B cell development and in peripheral B cell subsets. KLF2 is upregulated in pre-B cells as a consequence of pre-BCR signaling [15]. It is highly abundant in naïve follicular (FO) B cells, whereas only low levels of KLF2 can be detected in Marginal Zone (MZ) B cells [18,19,21]. In B1 cells, a specialized B cell subset located in the pleural and peritoneal cavities characterized by the secretion of natural IgM antibodies, high amounts of KLF2 can be found as shown by analyses of KLF2:GFP reporter mice [21,22]. Moreover, KLF2 is expressed in subsets of memory B cells (Bmem) [23,80,81]. KLF2-deficiency in B cells results in the expansion of MZ B cells, the loss and alteration of B1 cells, and the reduction in the size and the numbers of PP concomitant with reduced natural serum IgA levels [18,19,21].

During their development, B cell precursors migrate from the bone marrow via the blood to the spleen or LN. The migration of B cells to these organs and their proper localization requires CCR7 and CXCR5 signaling [82]. The homing to GALT is mediated by Itgα4β7 combined with CCR9- and CCR10-mediated chemotaxis [33]. The positioning of B cells in the B cell follicles of the lymph nodes and the spleen is mediated by CXCR5/CXCL13. Upon the T cell-dependent stimulation of naïve B cells (i.e., with a protein antigen) in LN or the spleen, B cells undergo immunoglobulin (Ig) class switch recombination, somatic hypermutation of their Ig genes and affinity maturation of their B cell receptors during the so-called germinal center (GC) reaction [83]. The GC reaction results in the generation of class-switched memory B cells with high affine BCRs and plasmablasts/plasma cells (PB/PC) that secrete high affine antibodies [83]. Initially, KLF2 is expressed in naïve FO B cells, then downregulated upon B cell activation and re-expressed in subsets of Bmem [23,80,81]. For PB there is evidence that their exit from the spleen to the blood stream is mediated, in part, by chemotaxis to S1P. Kabashima et al. demonstrated that both KLF2 and S1PR1 are upregulated in PB in the blood, indicating that KLF2 might have a functional role for their migration [52]. The shear forces in the bloodstream might trigger the upregulation of KLF2 in the circulating PB, similar to the shear, stress-induced KLF2 expression in endothelial cells [84], and thus trigger the expression of homing receptors. Importantly, KLF2 plays a role in the homeostasis of PB/PC. As shown by Winkelmann et al., TNP-specific IgG PC were reduced in the BM upon immunization in B cell-deficient KLF2 mice, indicating that KLF2 regulates the entry of PB to the BM or their survival within the BM niches (Table A1) [19]

As mentioned before, B cell-specific KLF2-deficient mice have enlarged spleens with an expansion of FO B cells and a predominant increase in MZ B cells [18,19,21] (Table A1). Along this line, the mutations of KLF2 in humans are involved in the generation of splenic marginal zone B cell lymphomas (SMZBL) [8,10,18,19,21] (Table A1). KLF2-deficient FO B cells exhibit a loss of L-Selectin and Itgβ7 expression. Both, L-Selectin and Itgβ7 are regulated by the direct binding of KLF2 to the respective promoters (Figure 1 and Figure 2) [58,61]. Interestingly, S1PR1 expression is not altered in KLF2-deficient FO B cells [19,21], indicating that S1PR1 expression is regulated independently of KLF2 in FO B cells. Along this line, MZ B cells which express only low amounts of KLF2 still express S1PR1 on their cell surface. In terms of migration, MZ B cells are characterized by shuttling between the MZ zone and the B cell follicle, as their functions include antigen uptake and delivery to the follicular dendritic cells (FDCs) in the light zone of GC [85]. Therefore, MZ B cells exhibit an alternating expression of CXCR5 (targeting them to the B cell follicles) and S1PR1 (targeting them to the marginal zone) to enable their shuttling between the marginal zone of the spleen and the B cell follicles. This shuttling process is directed by gradients of S1P and CXCL13 [85]. The observed low amounts of KLF2 in MZ B cells might enable the temporary upregulation of CXCR5 (similar to the mechanism found in TFH cells) which allows them to enter B cell follicles. How S1PR1 expression is regulated in MZ B cells remains unclear. One study claimed that CXCR5 and S1PR1 were downregulated in KLF2-deficient MZ B cells and upregulated in KLF2-deficient FO B cells [18]. However, these observations were in contrast to findings by other groups who reported that S1PR1 and CXCR5 expression remained unaltered in KLF2-deficient FO B cells [19,21].

The observed downregulation of L-Selectin and Itgβ7 on the surface of KLF2-deficient FO B cells might impair the entry of B cells into the LN and might be causative for the smaller sizes and lower cellularity of Peyers’ patches in KLF2-deficient mice [19,21]. As Itgβ7 is also required for the homing to the lamina propria (LP) of mucosal tissues, future studies will show whether KLF2 is involved in the regulation of IgA^+^ B cell trafficking to or within mucosal tissues.

KLF2 expression was also found in subsets of Bmem. Batthacharya et al. observed KLF2 expression in Bmem similar to that in naïve B cells. They found a correlation between KLF2 expression and the elevated expression of migration factors such as Itgβ7, S1PR1 and CCR6 [23]. Varying abundances of KLF2 transcripts in Bmem subsets which were defined by differential CD80 and PD-L2 expression were reported by Zuccarino-Catania et al. [81] Moreover, a recent publication by Riedel et al. demonstrated the existence of six different isotype-switched Bmem clusters by single-cell RNAseq with one cluster of KLF2-expressing Bmem [80]. The function of KLF2 in Bmem, however, remains elusive. KLF2 might promote Bmem survival, as well as Bmem migration and tissue retention. Based on the findings of Winkelmann et al. that IgG PC are virtually absent in the BM of KLF2-deficient mice upon boost immunization, together with the observation that KLF2 is expressed in PB in the blood [19,52], it is tempting to speculate that KLF2 modulates PB and PC trafficking, as well as their entry to survival niches in the gut and in the BM. Moreover, the observation that KLF2 is associated with specific, subsets of Bmem cells, suggests a regulatory function of KLF2 in the homeostasis of Bmem cell migration, survival and/or tissue residency.

## 6. KLF2 and Myeloid Cell Migration

Aside from its role in lymphocyte activation and migration, KLF2 is also expressed in cells of a myeloid origin. KLF2 is highly abundant in monocytes and is shown to be reduced upon activation, during the differentiation to macrophages, and during the generation of osteoclasts [86,87,88]. Mice that were hemizygous for KLF2 exhibited an increased expression of pro-inflammatory factors such as MCP-1 and Cox-2 in BM-derived monocytes combined with a higher recruitment of CD11b^+^/F4/80^+^/Ly6C^+^ monocytes in the blood and the peritoneal cavity. Moreover, KLF2-hemizygous mice showed an enhanced osteoclast differentiation and exhibited higher numbers of TRAP-positive osteoclasts in arthritic bone tissue. These findings implicated an important role for KLF2 in the control of inflammatory diseases (Table A1) [88]. The increase in the release of pro-inflammatory factors was confirmed in mice with myeloid-specific deletion of KLF2 [89]. The deletion of KLF2 in all myeloid cells by the use of the Lyz2-cre deleter identified KLF2 as a central regulator of innate immune responses to bacterial infections and septic shock (Table A1) and connected KLF2 expression to hypoxia, HIF-1α and NF-κB signaling networks. KLF2 inhibited the expression of HIF1-α through the inhibition of NF-κB signaling in LPS-activated macrophages by modulating the binding of the NF-κB co-activators p300 and P300/CBP-associated factor (PCAF) to the HIF-1α promoter [90]. 

The ectopic expression of KLF2 in LPS-activated monocytes resulted in reduced phagocytosis and inhibited the production of cytokines and chemokines such as IL-1β, IL-8, TNFα, MCP-1/CCL2, macrophage inflammatory proteins, CD40L, and tissue factor. Moreover, in vivo studies revealed that KLF2 fosters the recruitment of monocytes to inflamed sites [24,88]. The lentiviral overexpression of KLF2 mimicked the effect of simvastatin in the human THP-1 monocytic cell line and led to the downregulation of MCP-1 and tissue factor [91]. Accordingly, a KLF2 deficiency resulted in the induction of CCL2/MCP-1, CCL5, IL-6, and Cox-2 in the inflammatory, M1-type peritoneal macrophages [89]. An important role of KLF2 in macrophages was demonstrated by Roberts et al. who showed that KLF2 and KLF4 were involved in the regulation of apoptotic cell clearance by tissue-resident macrophages [92], an important feature to prevent autoimmunity.

Mechanistically, KLF2 might interfere with the NF-κB-signaling pathways by interacting with cofactors that are crucial for NF-κB activation. For example, a direct interaction of KLF2 with PCAF and p300 was observed [86]. The interaction leads to the removal of PCAF from NF-κB complex. Thereby, one could envision that KLF2 inhibits NF-κB signaling. Along this line, PCAF, but not p300, overexpression rescued cells from KLF2-mediated NF-κB inhibition [86].

Another interesting finding connects KLF2 function in macrophages to atherosclerosis. By using myeloid-specific, KLF2-deficient mice, Lingrel et al. showed that macrophages from those mice displayed an increased adhesion to endothelial cells. In addition, KLF2-deficient neutrophils showed a stronger adhesion to endothelium. By crossing a myeloid KLF2-deficient mouse line (LysM-cre KLF2fl/fl mice) with a low-density lipoprotein receptor (Ldlr)-deficient mouse line, a functional role for KLF2 in the course of atherosclerosis was uncovered (Table A1). Under a high-fat diet, the double-deficient mice showed increased signs of disease [93]. In support of these findings, KLF2-hemizygous mice on an Apolipidprotein-E (ApoE)-deficient background also showed increased, diet-induced atherosclerosis [94] (Table A1).

Furthermore, KLF2 plays a role in regulating the expression of CCR5 and MerTK chemokine receptors in intramuscular monocytes (shown in LysM-cre KLF2 fl/fl mice with myeloid cell-specific deletion of KLF2). The ligands of CCR5 (CCL4 and CCL5) are found to be upregulated in the injured muscles of mice with a myeloid deletion of KLF2. CCR5 induction plays an important role in resolving inflammatory processes such as arthritis [95] (Table A1). The increased expression of CCR5 and increased numbers of CCR5^+^ monocytes were found in injured muscles of myeloid-specific, KLF2-deficient mice. Therefore, KLF2 might contribute to monocyte recruitment after muscle injury. An important regulator in the context of resolving injuries is MerTK, a receptor tyrosine kinase known to be involved in the resolution of inflammation during injuries. MerTK is functionally involved in the regulation of phagocytosis. In myeloid-specific KLF2-deleted mice, the increased numbers of MerTK macrophages can be detected with a higher phagocytic activity compared to wt control mice [95] (Table A1).

Moreover, Nayak et al. proposed a regulatory function of KLF2 in thrombosis. KLF2-deficient neutrophils showed a decreased rolling, which coincided with a decrease in P-Selectin and tissue factor production (Table A1) [96]. 

Importantly, KLF2 was described to have a functional role in asthma. Zhu et al. showed that KLF2 was implicated in the migration of blood neutrophils in asthma patients and in an animal model. Asthma patients had neutrophils with a reduced KLF2 expression. Consequently, CXCR1 and CXCR2 receptors are upregulated. These observations were confirmed mechanistically in vitro, by showing that KLF2-knockdown resulted in the upregulation of CXCR1 and CXCR2 and, consequently, a higher migration rate [97]. Thus, reduced amounts of KLF2 in neutrophils promoted the severity of asthma by enhancing neutrophil migration involving CXCR1 and 2 upregulation (Figure 1, Table A1). Therefore, KLF2 might be useful as a predictive marker of asthma.

To determine the functional role of KLF2 in dendritic cell (DC)/T cell interaction, Alberts-Grill et al. analyzed mice with a DC-specific deletion of KLF2 mediated by CD11c (Itgax)-cre. KLF2-deficient DC exhibited higher expressions of CD86 and CD40 upon LPS-activation. Their ability to trigger T cell proliferation and cytokine production, as well as apoptosis, was enhanced. The loss of KLF2 in DC was also related to increased atherosclerosis (similar to the findings by Lingrel et al. using LysM-cre deletion of KLF2 [93]) in a transfer model using Ldlr-deficient mice as recipients for BM from DC-KLF2-deficient mice. Surprisingly, these mice exhibited leukopenia with a loss of T cells [98].

## 7. KLF2 and NK Cell Migration

Aside from its involvement in lymphocyte and myeloid cell activation, migration and adhesion, KLF2 is important for natural killer (NK) cell migration and tissue residency. During the maturation of NK cells, the homing of NK cells to the lymph nodes is controlled at least in part by the Foxo-1 transcription factor and the regulation of L-Selectin [99]. Foxo-1 is an important regulator of KLF2 activity. Foxo-1-phosphorylation induced by AKT targets Foxo-1 from the nucleus to the cytoplasm, thus preventing and inhibiting the activation of the KLF2 promotor [100]. The downregulation/repression of KLF2 as a consequence of PI3K/AKT-mediated Foxo-1 phosphorylation is a common regulatory mechanism also found in T cells and in B cells. In B cells, KLF2 mRNA is reduced in Foxo-1-deficient mice [101].

Comparative single-cell RNA-seq transcriptome profiles of human and murine NK cells, as well as blood and splenic NK cells studied by Crinier et al., revealed a higher abundance of KLF2, as well as Jun and Fos transcripts, in splenic NK cells compared to blood NK cells. Moreover, KLF2 was among the 37 shared genes that were expressed in human and mouse splenic NK cells [102].

KLF2-deficiency in NK cells achieved by vav-cre and T2-cre deleters resulted in the increased proliferation of NK cells and a reduced survival. In addition, KLF2-deletion led to a change in the expression of homing receptors resulting in an incapability of KLF2-deficient NK cells to reach IL-15 rich microenvironments/tissues. Typically, NK cells are in circulation and are capable of rapidly reacting to chemokine gradients to reach their target tissues. Under “normal” conditions, NK cells express L-Selectin, CX3CR1 and S1PR5 (encoded by *Edg8*). Under inflammatory conditions, NK cells express CCR2, CCR5 and CXCR3. Upon the tamoxifen-induced deletion of KLF2 using the T2-cre system, L-Selectin, CX3CR1 and S1PR5 (*Edg8*) expression were downregulated in NK cells. In contrast, CCR7 was upregulated upon KLF2 deletion [103] (Figure 1). However, KLF2 deletion did not alter the expression of the inflammatory homing receptors, CCR2, CCR5 and CXCR3. Similar to the mechanisms found in T cells (and B cells), KLF2 was downregulated in NK1.1- and NKG2D-activated NK cells through a PI3K-dependent pathway. In summary, KLF2 is crucial for NK cell survival and the recruitment of NK cells to microenvironments with high IL-15 abundances. Moreover, tissue-resident CD69^+^/CD16^−^ NK cells from human lungs were characterized by a low expression of L-Selectin, S1PR5 and KLF2, as well as KLF3. Subsets of tissue-resident NK cells were capable of producing IFNγ, TNF, MIP-1ß, GM-CSF and responded to IL-15 stimulation with elevated perforin and granzyme B production [104]. These findings confirmed the importance of the regulatory KLF2/L-Selectin/S1PR5 axis for NK cell localization and function.

## 8. Conclusions

In summary, KLF2 is a central regulator of a multitude of cellular processes, such as activation, proliferation, differentiation, cell fate decisions and migration. The deregulation of KLF2 results in profound alterations of B cell, T cell, NK cell and myeloid cell subsets with regard to their localization and function. Despite the presence of cell type-specific and tissue-specific regulations of KLF2 and its target genes, common regulatory axes in different immune cells exist, including L-Selectin and members of the S1PR receptor family. Moreover, the activation and repression of KLF2 activity follows a common pattern and is mainly controlled by Foxo-1 activity. Importantly, the gradual modulation of KLF2 abundance is decisive for the orchestrated activation or repression of its target genes and for cell fate decisions. The cofactors which modulate KLF2 to either act as a transcriptional activator or repressor are yet to be determined.

As summarized in Table A1, the deregulation of KLF2 contributes to a multitude of diseases. Hence, the identification of factors which control KLF2 function, as well as the elucidation of KLF2-regulated genes, will lead to a deeper understanding of disease-causing and disease-related molecular networks.

The emerging field of single-cell RNAseq analyses and the concomitant characterization of novel subpopulations has already identified KLF2 as a differentially regulated transcription factor in subsets of Bmem and NK cells. Hence, the functional characterization of KLF2-regulated target genes and signaling pathways will be a challenging task for future research.

## Data Availability

Not applicable.

## Appendix A

**Table A1:** Involvement of KLF2 in selected diseases.

Disease	Involvement of KLF2	KLF2-Related Effects	Cell Type	Refs.
HIV-1 infection	KLF2 downregulation -> S1PR1 and L-Selectin downregulation/CD69 upregulation	Defective CD4^+^ T cell migration	CD4^+^ T cells	[76,77]
Autoimmunity	KLF2 knockout (Foxp3-cre deleter)	Signs of psoriasis, signs of IBD	iTregs	[69]
Type1 Diabetes (Autoimmunity)	microRNA-92a regulating KLF2 -> dysregulated TFH precursor pool size	Autoimmunity against insulin producing beta-cells	TFH	[78]
Allergic Asthma	Notch knockout (CD4-cre deleter) -> downregulation of KLF2 -> downregulation of S1PR1	Th2 cells are primed to leave the lymph node and invade the lung by KLF2-mediated upregulation of S1PR1; KLF2 downregulation by Notch-deficiency prevents Th2 lung infiltration	Th2 cells	[79]
Asthma	reduced KLF2 expression in blood neutrophils of Asthma patients -> upregulation of CXCR1 and CXCR2	Enhanced neutrophil migration	Neutrophils	[97]
Arthritis	Hemizygous KLF2 mice	Increased pro-inflammatory factors, osteoclast differentiation	Monocytes, Osteoclasts	[88]
Marginal Zone B cell Lymphoma	KLF2 knockout (mb1-cre deleter, CD19-cre deleter) & KLF2 mutations in human SMZBL -> strongly enriched MZB pool size	Splenomegaly (mouse),Marginal Zone B cell Lymphoma (human)	B cells	[8,10,18,19,21]
Atherosclerosis	KLF2 knockout (LysM-cre deleter) on Ldlr-deficient background; hemizygous KLF2 on ApoE-deficient background	Increased adhesion to endothelial cells, Increased signs of atherosclerosis when combined with Ldlr-deficiency or ApoE-deficiency	Macrophages/Neutrophils	[93,94]
Atherosclerosis	KLF2 knockout (CD11c-cre deleter) -> CD86 and CD40 upregulation upon LPS activation	Enhanced T cell activation, Increased signs of atherosclerosis when combined with Ldlr-deficiency	Dendritic cells	[98]
Thrombosis	KLF2 knockout (LysM-cre deleter) -> decreased P-Selectin	Decreased rolling ability	Neutrophils	[96]
Muscle Injury	KLF2 knockout (LysM-cre deleter) -> CCR5 upregulation	Increased numbers of CCR5^+^ monocytes and of MerTK^+^ macrophages in injured muscles -> increased phagocytosis activity	Macrophages	[95]
Sepsis	KLF2 knockout (Lyz2-cre deleter)	Endotoxic shock	Myeloid cells	[90]
